# Advanced imaging techniques for neuro-oncologic tumor diagnosis, with an emphasis on PET-MRI imaging of malignant brain tumors

**DOI:** 10.1007/s11912-021-01020-2

**Published:** 2021-02-18

**Authors:** Wynton B. Overcast, Korbin M. Davis, Chang Y. Ho, Gary D. Hutchins, Mark A. Green, Brian D. Graner, Michael C. Veronesi

**Affiliations:** 1 Department of Radiology and Imaging Sciences, Indiana University School of Medicine, 550 N University Blvd. Room 0663, Indianapolis, IN 46202 USA; 2 Department of Radiology and Imaging Sciences, Indiana University School of Medicine, Goodman Hall, 355 West 16th Street, Suite 4100, Indianapolis, IN 46202 USA; 3 Department of Radiology and Imaging Sciences, Indiana University School of Medicine, Research 2 Building (R2), Room E124, 920 W. Walnut Street, Indianapolis, IN 46202-5181 USA; 4 Department of Radiology and Imaging Sciences, Indiana University School of Medicine, Research 2 Building (R2), Room E174, 920 W. Walnut Street, Indianapolis, IN 46202-5181 USA

**Keywords:** Brain tumor, Advanced MRI, Amino acid PET, FET, Hybrid PET/MRI, Radiogenomics, Glioma, Glioblastoma, Metastasis, High-grade malignancy, Progression, Pseudoprogression, Radiation necrosis, Pseudoresponse, Treatment-related change, Tumor grading, Perfusion-weighted imaging, Diffusion-weighted imaging, Chemical exchange saturation transfer, MR spectroscopy, Radiomics

## Abstract

**Purpose of Review:**

This review will explore the latest in advanced imaging techniques, with a focus on the complementary nature of multiparametric, multimodality imaging using magnetic resonance imaging (MRI) and positron emission tomography (PET).

**Recent Findings:**

Advanced MRI techniques including perfusion-weighted imaging (PWI), MR spectroscopy (MRS), diffusion-weighted imaging (DWI), and MR chemical exchange saturation transfer (CEST) offer significant advantages over conventional MR imaging when evaluating tumor extent, predicting grade, and assessing treatment response. PET performed in addition to advanced MRI provides complementary information regarding tumor metabolic properties, particularly when performed simultaneously. ^18^F-fluoroethyltyrosine (FET) PET improves the specificity of tumor diagnosis and evaluation of post-treatment changes. Incorporation of radiogenomics and machine learning methods further improve advanced imaging.

**Summary:**

The complementary nature of combining advanced imaging techniques across modalities for brain tumor imaging and incorporating technologies such as radiogenomics has the potential to reshape the landscape in neuro-oncology.

## Introduction

Primary central nervous system tumors have one of the leading cancer incidence rates across patients of all ages with nearly 400,000 US cases reported between 2012 and 2016 [1]. Greater than two-thirds are considered benign; the remaining malignant tumors contribute to significant morbidity and mortality [2]. Secondary brain tumors, including brain metastases, are ten-fold more frequent and have a major impact on outcome data [3]. Although significant advancements have been made in the management of brain tumor patients, high-grade malignancies remain difficult to treat. For instance, the life expectancy of patients with glioblastoma (GBM) was around 20 months with addition of tumor-treating fields to the standard of care therapy regimen [4]. More broadly, only 23.5% of patients with a malignant brain or spine tumor are expected to still be alive after 5 years, based on data compiled from the Central Brain Tumor Registry between the years of 2013–2017 [5].

The critical role of advanced imaging in the diagnosis, treatment, and subsequent management of brain tumors is discussed in this review. Conventional MRI is useful for initial assessment, but can present limitations when evaluating tumor extent, predicting grade, and assessing treatment response. Advanced MRI techniques such as perfusion-weighted imaging (PWI), MR spectroscopy (MRS), diffusion-weighted imaging (DWI), and MR chemical exchange saturation transfer (CEST) are helping address this shortcoming. PWI shows great potential, especially for determining tumor progression versus treatment related change, but standardization is needed across institutions. While MRS has shown high accuracy in publications, clinical application poses challenges. Diagnostic confidence can be improved when combining with additional MR sequences and modalities. DWI can contribute to tumor characterization in select circumstances. Newer quantitative diffusion tensor imaging (DTI) techniques that measure the degree of cellularity are in development. CEST is still in the advanced clinical research and development phase. CEST has great potential for patients who cannot receive MRI contrast since it relies on intrinsic properties of mobile phase proteins in the cell. PET imaging provides complementary information regarding tumor metabolic properties and can be performed with or combined with advanced MRI. Amino acid PET is emerging as the key to diagnostic specificity. The overall direction of amino acid PET imaging favors the use of ^18^F-fluoroethyltyrosine (FET) given its additional well characterized properties for both static and dynamic tumor imaging. The typical conclusion of publications that utilized separately obtained PET and MRI points to the superiority of hybrid PET/MRI systems. Recent papers using hybrid PET/MRI systems highlighted in this review strengthen these conclusions. Finally, we briefly discuss a complementary role for radiogenomics in neuro-oncologic disease management.

## Magnetic resonance imaging

### Conventional MRI

For many years, conventional MRI has been the standard modality to diagnose and localize brain tumors, perform stereotactic biopsy, plan surgical resection, and distinguish post-treatment changes from recurrent tumor. Conventional MRI sequences include pre- and post-contrast T1-weighted, T2-weighted, T2-based fluid attenuation inversion recovery (FLAIR), DWI with apparent diffusion coefficient (ADC), and either susceptibility-weighted or gradient echo imaging. The Response Assessment in Neuro-Oncology (RANO) working group has defined many of the metrics for tumor analysis in conventional MRI. Measurable disease requires a bi-dimensional measurement of a contrast enhancing lesion with margins that are clearly discernable. The measurements should be perpendicular and at least 10 mm in length, visible on at least two axial slices. Non-measurable disease applies to lesions that are less than 10 mm have unclear surgical margins or where only a single dimensional measurement can be made. In 2010, the RANO group added non-enhancing mass-like T2/FLAIR signal as part of the non-measurable criteria [6]. The RANO criteria has more limited application when discerning true tumor extent or tumor progression from treatment-related changes, which includes pseudoprogression, radiation necrosis, and pseudoresponse [7]. Extensive work is being done to overcome limitations of conventional imaging. Development of PWI, MRS, DWI, and CEST are discussed in the next section.

### MR Perfusion-weighted Imaging

PWI is a technique that measures tumor vascularity by dynamically evaluating tissue after either exogenous contrast administration or through non-invasive labeling of endogenous water molecules. The three main perfusion techniques available clinically include dynamic susceptibility contrast (DSC), dynamic contrast enhancement (DCE), and arterial spin labeling (ASL). The most used technique, DSC, takes advantage of signal loss amplification inherent in susceptibility-weighted sequences with passage of paramagnetic gadolinium through the tissue. The most commonly assessed DSC parameter is relative cerebral blood volume (rCBV), which is increased in tumors secondary to microvascular density and slow flowing collateral vessels. The cut off for differentiating tumor from non-tumor treatment related changes is without an international consensus. One meta-analysis reported rCBV ratio thresholds ranging from 1.49 to 3.10 [8]. In general, an rCBV ratio of greater than 2 favors either high-grade tumor or tumor progression over treatment-related change [9]. DSC limitations include poor spatial resolution, susceptibility artifact, and rCBV measurements influenced by blood–brain barrier disruption, as contrast is assumed to remain in the intravascular space [10, 11]. Multiple meta-analyses have demonstrated high accuracy (>90%) of DSC in delineating tumor recurrence from radiation necrosis [8, 12, 13]. DSC identified tumor neoangiogenesis as a biomarker which could discern between low-grade and high-grade gliomas [14, 15]. In addition, Law et al. demonstrated that DSC helped identify low-grade gliomas that progress rapidly or have higher propensity for malignant transformation [16]. In a prospective study, 13 patients were followed with DSC perfusion and tumors that transformed from low-grade to high-grade glioma demonstrated continuous increase in rCBV DSC up to 12 months before contrast enhancement became apparent [17]. In a multicenter phase II trial of bevacizumab with irinotecan or temozolomide, patients were imaged with DSC and rCBV values recorded at 2, 8, and 16 weeks after treatment initiation [18]. An early decrease in rCBV was predictive of improved survival when recurrent GBM was treated with bevacizumab. DSC further dissociated between isocitrate dehydrogenase (IDH) wild-type and mutant gliomas [19] and characterized additional molecular and genomic profiles along with textural analysis [20].

DCE MRI utilizes the T1 relaxivity of gadolinium contrast, which is superior to DSC in the presence of hemorrhage. DCE perfusion generates quantitative parameters within a single volume of interest (VOI), including volumetric transfer constant (kTrans), fractional plasma volume (Vp), fractional volume of the extravascular extracellular space (Ve), and semi-quantitative parameters such as area under the curve (AUC). Although DCE has the potential to overcome certain shortcomings of DSC, the technique is less well studied than DSC, is used less frequently in clinical practice, and calculations rely heavily on model dependent approximations [21, 22]. Despite these challenges, DCE is effective in distinguishing between tumor recurrence and post-treatment changes, in particular radiation necrosis [8, 13]. For instance, in 79 patients with new or increasing contrast enhancing lesions following chemo-radiation, DCE distinguished pseudoprogression from true progression with a sensitivity of approximately 89% and specificity of 80% [23]. Despite the strengths and potential uses, further standardization is needed before DSC and DCE can be considered a viable strategy across institutions.

ASL is performed using a radiofrequency pulse which labels endogenous water molecules in blood vessels, which can subsequently be measured as signal reduction as they pass through the tissue of interest. Use of ASL is compromised by signal-to-noise ratio, but has still been shown to identify recurrent disease from radiation necrosis [24]. ASL is also advantageous, as no exogenous contrast is needed. The technique may continue to improve with increasing magnet strengths but currently is used much less frequently than DSC or DCE [24, 25].

### MR Spectroscopy

MRS is an analytical method used to non-invasively assess for water-soluble brain metabolites according to their precession frequency. Since MR induces a characteristic magnetic field in nuclei of differing number of electrons, these various signature resonant frequencies can be detected and analyzed. The chemical species frequently assessed on MRS include creatinine (Cr), N-acetyl aspartate (NAA), choline (Cho), lactate, lipids, alanine, glutamine, glutamate, 2-hydroxyglutarate, citrate, and myoinositol. The Cr peak is utilized as an internal reference standard relative to other peaks since its level remains high and is relatively comparable across different brain tissue types [26]. Choline is a marker of cell proliferation given its presence in the cell membrane [27, 28]. NAA is found exclusively in neurons and can define cell density and cell viability, [29–31]. Other metabolites can measure tumor metabolism (glucose) and necrosis (lactate or lipids) [32]. Lactate is a marker of anaerobic glycolysis and may represent hypoxic adaptation in higher grade tumor tissue [33]. Myoinositol is a marker of gliosis which can be increased in low-grade gliomas [34, 35]. 2HG is an oncometabolite that is an important biomarker for glioma with IDH mutations, and it can predict tumor grade, tumor progression and the likelihood of treatment response [36].

The standard MRS technique options include single or multi-voxel analysis. With single voxel spectroscopy (SVS), the average metabolite concentration is measured across the volume of a chosen region of interest. SVS at 3T with good B0 homogeneity should provide diagnostic quality spectra from tissue volumes down to 4 cm^3^ over a 5-min acquisition time [37]. Advantages of single voxel MRS are ease of use, shorter scan time, greater field homogeneity, and higher signal to noise ratio compared to multi-voxel spectroscopic imaging. The drawbacks of single voxel spectroscopy include lower spatial resolution across a larger component of tissue, fixed grid placement, and subjective variability of VOI placement. The multi-voxel technique overcomes limitations of single voxel spectroscopy by providing a grid of multiple voxels over a larger region of interest and flexibility of voxel repositioning during post-processing. A multi-voxel spectrographic (MVS) acquisition using a 16 × 16 grid of spectra at 3T with 1.5 cm^3^ voxel resolution at TR 1500 ms, an average of one phase encoding step, and elliptical *k*-space sampling may be acquired over an approximately 5-min time period [37]. MVS imaging suffers from lower signal-to-noise ratio and cross-voxel signal contamination which impedes precision of quantitation. Additional concerns regarding MRS in general are lack of standardization of imaging acquisition, differences in magnet strength, and artifact induced by either the presence of blood hemosiderin within a tumor or marrow lipid within the calvarium for more peripheral lesions [38]. To overcome inherent limitations of both single and multi-voxel spectroscopy, other techniques are being developed. For instance, Li et al. developed an innovative super-resolution whole brain three-dimensional spectroscopy technique to visualize 2HG which had resolution equal to the best single voxel spectroscopy (0.32 cm^2^) [39]. The technique also would eliminate operator dependence and detect potential tumor regions outside of standard two-dimensional spectroscopic regions of interest. The main drawback to this technique was long scan times of greater than 18 min for a single acquisition.

MRS was reported to have high accuracy (92%) for differentiating neoplastic from non-neoplastic tissue and was improved when combination with other advanced MR imaging such as PWI (96%) [40]. Various studies since have also shown similar accuracy [13, 41, 42], but a meta-analysis indicated that MRS may have more benefit when combined with other advanced imaging modalities rather than interpreting the results with MRS alone [43]. The benefit of combining various imaging modalities has been recommended in more recent papers as well [44–46]. Higher Cho and lower NAA with increased lactate are more consistent with glioma rather than metastases or central nervous system lymphoma [47, 48]. Although there is no standardized numerical cutoff value for these metabolites, a Cho/NAA of 2.2 is proposed as a reasonable cutoff [38]. For glioma grading, MRS was better than conventional imaging alone [49]. Using Cho/NAA and Cho/Cr ratios, an accuracy of 80–97% was achieved in distinguishing tumor progression from radiation necrosis [50, 51]. The most heralded use of MRS in the presence of a known malignant brain tumor has been for distinguishing progression of tumor from treatment related MRI changes (i.e., pseudoprogression). However, in reality, differentiating these two entities remains highly challenging since both processes can present with low NAA from neuronal loss and dysfunction, high Cho from abnormal cellular membrane attenuation/integrity, and high lactate and lipids from anaerobic metabolism [52].

### MR Diffusion-weighted Imaging

Along with its routine clinical role, DWI provides important functional and physiological information about brain tumors and the peri-tumoral microenvironment. Direct measurement of water mobility becomes an imaging biomarker of tissue pathology, as its movement is dependent on factors such as viscosity, cellularity, and the tortuosity of the extracellular space [32]. The ADC sequence provides quantitative data derived by measuring restriction of water molecules at differing degrees of diffusion weighting (i.e., *b* = 0, 500, 1000, or even 3000 s/mm2). At least two different *b* values are required, and the ADC value for each voxel is calculated using linear regression. Restriction of water diffusion secondary to tumor cellularity results in hypointense or low ADC values, useful for differentiating tumor type and grade [53, 54]. For instance, the degree of ADC hypointensity will typically be greater in lymphoma, a highly cellular tumor, as compared to high-grade glioma or metastases. Similarly, non-necrotic high-grade glioma and metastases will have greater reduction in ADC values than low-grade malignancies. Edema associated with high-grade tumors limits ADC sensitivity, as it increases the average ADC intensity [32].

DWI can also be useful for treatment response and differentiating chemo-radiation-induced changes from tumor given lower ADC signal of tumor [55]. Restricted diffusion in the postsurgical setting helps identify cytotoxic edema. The associated parenchyma can enhance in the subacute setting and could be confused with tumor progression. Accordingly, baseline MRI is recommended within 48 h of surgery [56]. DWI has been shown to predict prognosis within three weeks of therapy initiation [57, 58] and was also predictive of treatment response to bevacizumab [59–61]. For treated GBM, progression of disease typically demonstrates lower ADC values compared with pseudoprogression. A meta-analysis demonstrated moderate accuracy using ADC to delineate progression of GBM from radiation necrosis with a pooled sensitivity of 71–82% and sensitivity of 84–87% [13, 62]. The proposed ADC threshold for disease progression in GBM is 1.3 × 10^−3^ s/mm^2^ [41, 63].

Standard DWI acquisition suffers from the assumption that water diffusion occurs in the absence of boundaries via a uniform Gaussian distribution. In reality, the structure of cell membranes, intracellular organelles, and water compartments in cerebral tissues make this assumption inaccurate [64]. Therefore, more sophisticated models of quantitative DWI have been developed, such as diffusion tensor imaging (DTI) [65]. In DTI imaging, the application of a diffusion tensor model onto DWI data determines diffusion along each of three axes of a given voxel and produces a three-dimensional ellipsoid, known as the diffusion tensor. The diffusion tensor provides more complete anisotropic and structural data of each voxel, yielding fractional anisotropy images which clearly delineate white matter tracts by coding left–right (red), anterior–posterior (green), and superior–inferior (blue) diffusion. Diffusion kurtosis imaging (DKI) extends the principles of DTI even further to quantify the deviation from a Gaussian distribution to create a more accurate model [66, 67]. By acquiring data in more than 15 nonlinear directions, both kurtosis metrics (mean, axial, and radial kurtosis) and conventional diffusion metrics (mean, axial, radial diffusivity, and fractional anisotropy) are obtained. Meta-analyses that examined the diagnostic accuracy of DKI for differentiating high- and low-grade gliomas projected a pooled area under the curve of 0.94 and 0.96 for mean kurtosis [68, 69]. More recently, Abdalla et al. performed an extended and updated meta-analysis and also concluded that DKI has good diagnostic accuracy in differentiating high- from low-grade gliomas [70]. Accuracy was consistent across different studies with varied acquisition and post-processing techniques, indicating DKI may be useful across different institutions and populations given greater optimization and standardization. Additional promising advanced DTI techniques include intravoxel incoherent motion (IVIM) and neurite orientation and dispersion imaging (NODDI), which utilizes multiband imaging [71]. IVIM allows separate estimates of tissue diffusivity and microcapillary perfusion, while NODDI can measure the microstructure of dendrites and axons to provide data on neuronal changes.

### MR Chemical Exchange Saturation Transfer

MR CEST is a non-contrast technique which detects and amplifies metabolic substrates in the tumor tissue not found with other MRI sequences [72–75]. Imaging relies on the exchange between targeted chemical compounds and bulk water [76]. The most frequent CEST utilized for brain tumor imaging is amide proton transfer (APT) also called amide-CEST MRI, which detects and amplifies an exchange between the intrinsic hydrogen protons located on amide groups and water molecules within tissue [77]. The most abundant source of amide groups in tissue are contained on the amino acids of mobile cellular peptides and proteins, which are elevated above background brain in various disease states, including brain tumors [78]. Therefore, amide-CEST MRI derives its signal specificity from an overabundance of cytosolic proteins within GBM cells. In addition to amide groups, hydroxyl and amine groups are also mobile protons that can be used to generate detectable CEST signals (i.e., amine-CEST and hydroxyl-CEST). In comparison to metallic contrast agents (i.e., gadolinium or iron oxide), CEST does not negatively impact the intrinsic MRI properties of tissues nor induces a tissue toxicity potential [76]. Although an advantage of CEST is the reliance on intrinsic contrast within the tissue, exogenous application of solutes such as glucose [79] and glucose derivatives [80] can also be utilized. Largely though, CEST has been developed to take advantage of intrinsic unique properties of peptide and protein detection in the mobile phase. Another CEST technique that has recently been applied to GBM detection is the relayed nuclear Overhauser effect (rNOE), located in the *Z*-spectrum up field from water resonance (0–5 ppm) [81, 82]. Both APT and rNOE signals are associated primarily with protein and peptide presence, although rNOE may be more associated with protein size and configuration [83, 84]. Therefore, it is likely that APT and rNOE provide different but similarly valuable information holding promise as individual imaging biomarkers for different tumor properties. Since the discovery and improvement of CEST, the technique has been applied to human imaging in a complementary manner to other MRI methods, particularly with regard to differentiating infiltration of tumor compared with peri-tumoral edema [85, 86]. In the clinical setting, amide-CEST MRI demonstrated an ability to delineate low- from high-grade gliomas [87–89], differentiate tumor from treatment-related changes including pseudoprogression [90, 91], predict treatment response of brain metastases following radiation [92], and provide early (2-week) imaging biomarker evidence of GBM response to chemo-radiation therapy [93]. Finally, amide-CEST MRI has potential for identifying IDH mutation status in low-grade tumors as well as MGMT methylation status in high-grade tumors [94, 95].

### MR Functional Magnetic Resonance Imaging and Diffusion Tensor Imaging

While previously discussed advanced MRI techniques provide valuable anatomic, molecular and chemical information, functional MRI (fMRI), and DTI are readily available cortical and white matter mapping techniques which provide valuable information on brain activity adjacent to brain tumors. Blood oxygen level–dependent (BOLD) fMRI relies on the paramagnetic properties of oxyhemoglobin, which is increased relative to deoxyhemoglobin in the activated regions of the brain during either task-based or resting-state acquisition. Task-based fMRI can be tailored to specific motor and language functions prescribed to delineate eloquent cortices in relation to tumor location. Resting-state fMRI evaluates neural networks at rest and can be a useful adjunct in cognitively impaired patients, especially for language mapping [96, 97]. fMRI results must be interpreted with caution, as they can be limited by susceptibility, respiration, medications, motion, task uniformity, tumor vascularity leading to neurovascular uncoupling, and patient cooperation [98–100]. In one study, clinicians anecdotally reported a 30% discrepancy between fMRI results and direct cortical stimulation mapping at surgery, although fMRI can map beyond the surgically exposed gyri [101]. DTI uses anisotropic diffusion properties of axonal water to delineate white matter tracts such that their relationship with the planned approach and resection can be identified. A 1-cm margin of safety from critical structures has been proposed and utilized [102, 103]. When employed in conjunction with other advanced MRI techniques, fMRI and DTI can help preserve eloquent regions of the brain while maximizing high-grade tumor resection volume.

## Positron Emission Tomography

PET imaging relies on detection of emitted photons from intravenously delivered positron emitting radiotracers to provide dynamic functional molecular imaging. Depending on the molecule the radionuclide is bound to, biological processes such as glucose consumption and amino acid/analogue uptake can be visualized non-invasively and quantitatively. A PET scanner detects emitted pairs of 511 keV photons through annihilation coincidence detection to obtain projections of radioactivity distribution in the patient. Most PET systems are coupled to CT scanners, although MRI coupled systems are increasing in number and are more optimal for brain tumor imaging. Typically, brain tumor PET results are based on standard uptake value (SUV) and reported as a mean and maximum tumor to brain ratio (TBR). Certain radiotracers reveal additional dynamic information through the study of radiotracer uptake and release characteristics, including time to peak and slope of the curve. Advantages of PET include high quantitation and sensitivity and the ability to co-register with other imaging modalities. Limitations of PET include lower spatial resolution, radiation exposure, and relatively high cost with complex equipment. While there are many new and experimental PET radiotracers in development, clinically available PET radiotracers for brain tumor imaging are comprised of mostly ^18^F-fluorodeoxyglucose (FDG) and amino acid radiotracers, which include FET, ^11^C-methylmethionine (MET), and 3,4-dihydroxy-6-^18^F-fluoro-L-phenylalanine (FDOPA).

### Fluorodeoxyglucose PET

The wide availability and low cost of FDG has led to many published studies in brain tumor imaging, although high physiologic background uptake is a significant limitation [104, 105]. Technique modifications have attempted to offset the difficulty of FDG to delineate tumor borders and overcome nonspecific uptake in other processes such as with inflammation. Multiphase FDG PET/CT was recently used in the evaluation of high-grade gliomas and metastatic lesions [106]. Normal brain FDG activity decreases slightly over time, while activity in malignant tissue remains steady or increases. This difference was exploited by fusing dual-phase CT with MRI. Others have combined FDG PET/CT with MR perfusion. In a prospective study, FDG and DCE were both useful in distinguishing between progression and radiation injury, although DCE slightly outperformed FDG [107]. ASL perfusion, DSC perfusion, and FDG PET/CT were studied in 30 patients with grades II–IV glioma previously treated with surgery and proton beam therapy for the ability of each modality to distinguish progression of disease from radiation necrosis [108]. In regions with mixed radiation necrosis, ASL was considered superior to DSC and FDG for detecting tumor recurrence.

Despite the limitations of using FDG in brain tumor detection, hybrid FDG PET/MRI appears to improve diagnosis. Hojjati et al. compared hybrid FDG PET/MRI and FDG PET/CT in 19 patients who underwent both exams in a single day [109]. Using a TBR mean of greater than 1.31, FDG PET/MRI was superior to FDG PET/CT with an AUC of 0.94. In another hybrid FDG PET/MRI study, 35 glioma patients with 41 lesions were assessed with suspected tumor progression or treatment-induced necrosis [110]. Individual parameters which achieved significance include rCBV mean (77.5%), ADC mean (78.0%), Cho/Cr (90.9%), TBR max (87.8%), and TBR mean (87.8%). When all three MR imaging parameters were combined, the AUC improved to 0.913. Similarly, hybrid PET/MRI was performed in 41 patients with high-grade glioma to compare FDG performance to DCE perfusion metrics [111]. FDG had lower specificity than DCE (56% vs. 89%), but the overall accuracy was similar (80% vs. 83%). There was up to 27% discordance between FDG and DCE. Pyatigorskaya et al. assessed the potential for FDG PET/MRI to overcome limitations of separately performed MRI and FDG PET to differentiate between recurrence of high-grade glioma compared with radionecrosis [112]. Combined PET/MRI analysis differentiated between recurrence and radionecrosis with improved diagnostic accuracy (95% vs. 63% for PET alone and 82% for MRI alone). FDG being a reliable, cost-saving radiotracer, combined FDG PET/MRI could still play a role in the follow-up of high-grade brain tumors where amino acid PET is not available.

### Amino Acid PET with an Emphasis on FET PET

To help address critical shortcomings of MRI and FDG PET, international working groups recommend amino acid PET imaging in conjunction with MRI for assessment of primary high-grade malignant brain gliomas [104] [113] and for brain metastases [105]. Amino acid PET is recommended for discerning neoplastic from non-neoplastic processes, delineation of tumor extent for resection or re-resection planning, hot spot localization for biopsy planning, prognostication, post-resection assessment, radiation therapy planning, baseline monitoring for chemo-radiation, diagnosis of treatment-related changes versus progression/recurrence, and baseline imaging for adjuvant treatment monitoring [104]. Numerous studies have been published on MET, FET, and FDOPA that substantiate these recommendations, and have been reviewed extensively, for instance by Werner et al. [114], Stegmayr et al. [115] and Lohmann et al. [116]. Although a promising amino acid agent, MET suffers from a short half-life (20 min compared with 110 min for FET and FDOPA), making production possible only in centers with a cyclotron [117, 118]. The advantages of F18 labeling and extensive clinical results for FET have largely replaced short lived radiotracers in many centers across western Europe, including MET [115]. For instance, using static and dynamic FET PET and conventional MRI, delineation between pseudoprogression and tumor progression has an accuracy of 96% (sensitivity 100% and specificity 91%) [119]. FDOPA is also a promising amino acid PET agent, but there is not nearly as much published data in comparison to FET, which has been used safely in humans since 1999. Using FDOPA it is also difficult to distinguish tumor involving the nigrostriatal structures as there is intrinsic physiologic uptake of the radiotracer in this region. The cost effectiveness of amino acid PET in Europe has also been demonstrated for differentiating recurrent brain metastasis from radiation induced enhancement [120].

**Fig. 1:**
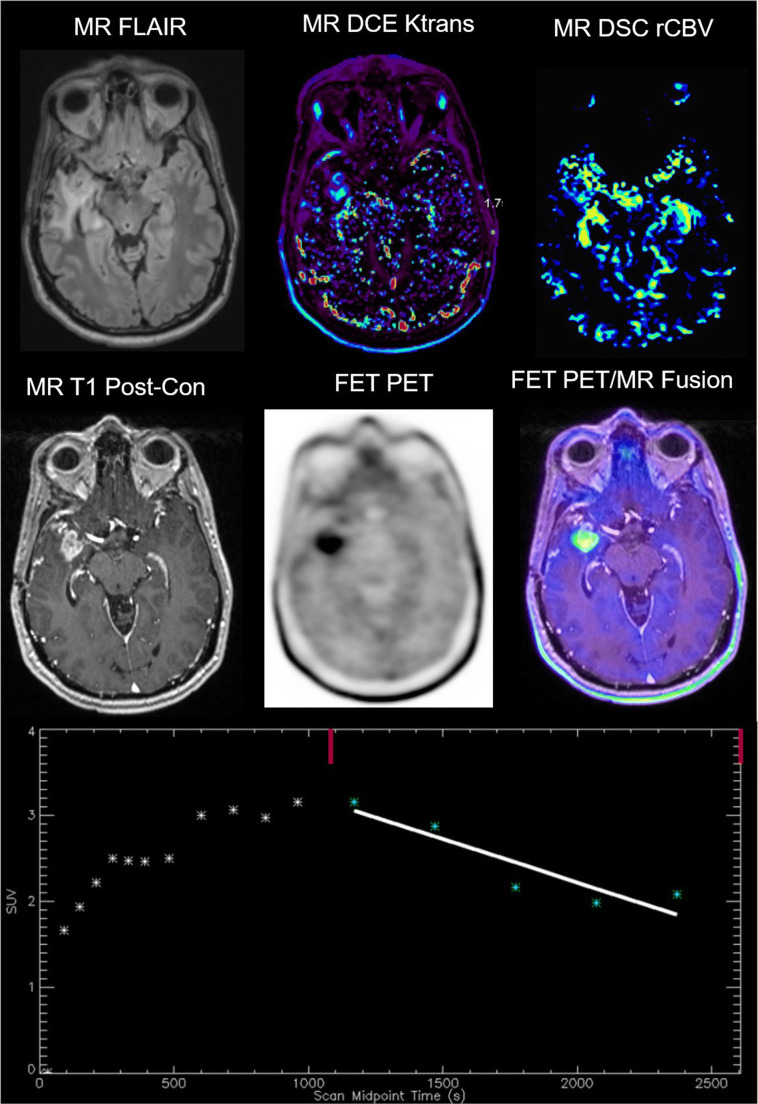
Hybrid FET PET/MRI imaging. Representative images of a WHO grade IV GBM with a lesion progressively enlarging at 6 months following surgery and radiation therapy. Top left to bottom right include FLAIR, DCE Ktrans, DSC rCBV, T1 post-contrast, FET PET, and fused hybrid FET PET/MRI images. Both DCE and DSC images show abnormal perfusion corresponding to areas of abnormal enhancement indicating increased vascularity. The bottom middle image shows abnormally elevated FET PET signal in a pattern consistent with the MRI perfusion findings. Subsequent surgical resection confirmed tumor recurrence on histopathology. TBR max was 3.74 and TBR mean was 3.13.The dynamic characteristics of the FET uptake can also be studied in a region of interest over time as shown on the time activity curve (*Y* axis = absolute SUV, *X* axis = time in seconds). In this case, a 45-min acquisition occurred. The slope of the curve was − 3.60. A downward slope at the second half of the acquisition is suggestive of presence of tumor, while an upward slope has been reported to be more indicative of treatment related change such as pseudoprogression. Data was generated in QImage Softwared, NIH NCI 5R01CA202695. Images were de-identified and approved under an institutional IRB protocol.

FET is a synthetic amino acid derived from tyrosine, and just like tyrosine, is passively taken into cells via the system L transporter (LAT) in exchange for leucine [121]. LAT1 and LAT2 are over expressed on the membrane of tumor cells, secondary to a need for amino acids for manufacturing protein [122]. The LAT receptors were also overexpressed in brain tumor cells undergoing rapid cell proliferation and in tumor angiogenesis [123]. Once inside the cell, FET is not associated with a known efflux transporter and is otherwise not metabolized further [121]. Therefore, the retention time in pathologic tumor tissue is higher than for other natural amino acid radiotracers. Although static amino acid PET parameters such as TBR max and mean have high diagnostic accuracy alone, addition of dynamic FET PET imaging may contribute to increased confidence of diagnosis. Unpublished imaging at our institution shown in Fig. 1 mirrors characteristic dynamic FET features [124–126] with more rapid tracer uptake into higher grade tumor tissue (early upslope) and subsequent wash-out (late downslope).

Combination of individually acquired FET PET with MR PWI in assessment of high-grade glioma has shown promise for determining glioma extent and grading and assessing tumor progression from pseudoprogression. FET PET also exhibits high diagnostic accuracy in differentiating radiation injury from tumor progression in patients with brain metastases [124, 127, 128]. Recurrence prediction for GBM was assessed with FET PET/CT and compared with FDG PET/CT in 16 patients who also underwent DWI and DCE perfusion [129]. While FDG, FET, and PWI correlated in both contrast enhancing and non-contrast enhancing tissue that progressed, FET proved to be the most important parameter for predicting recurrence based on standardized coefficients for recurrence models.

Individual FET PET subsequently combined with MRS exhibit improved sensitivity and specificity when used in a complimentary manner versus either modality alone [130–132]. Both FET PET and MRS were employed in 50 patients with newly diagnosed suspected diffuse gliomas (167). Both FET TBR and NAA/Cho ratio were independently predictive of histologic identification of tumor tissue. While MRS had higher sensitivity, FET TBR exhibited higher specificity. When both FET PET and MRS were positive, an accurate diagnosis of tumor was determined in 97% of patients as validated histologically. An emerging theme is spatial incongruence between MRS and PET. In a study by Mauler et al., PET and MRS were incongruent based on their respective mean center of the mass by 9 ± 8 mm [133]. In another a study to assess spatial congruence between FET PET and MRS, TBR was compared to concentrations of Cho, Cr, and total NAA in 15 patients with grades II–IV gliomas [134]. Only an elevated Cho/NAA ratio correlated with strong or moderate elevation in FET TBR.

FET PET/CT has also been assessed against DWI and CEST. Data from initial studies revealed incongruent results, suggesting FET, DWI, and CEST evaluate different biologic processes and information in brain tumors [135, 136]. However, a more recent study from 2020 utilized FET PET-MRI combined with amide-CEST and DSC perfusion to generate intra-lesional hot spot volumes (HSV) in 46 glioma patients [137]. HSVs generated with amide-CEST in GBM patients were larger than for FET or DSC HSVs. Amide-CEST HSVs were lower in patients with low-grade gliomas compared with GBM patients. In addition, there was a high correlation of the HSVs for amide-CEST and FET regions. When compared with tissue from targeted biopsies in 10 GBM patients, both amide-CEST and FET correlated significantly with cellularity while tissue vascularity correlated with CBV and FET.

### Hybrid FET PET/MRI

Of all studies to date on hybrid PET/MRI, FET has been utilized the most, further confirming its utility in brain tumor imaging. In a study by Filss et al., hybrid FET PET/MRI was performed to compare FET TBR with DSC rCBV values in 56 patients with gliomas, including 24 patients with GBM [138]. The most important finding was that tumor volumes were significantly larger using FET TBR maps compared to rCBV maps. Spatial overlap of both imaging parameters was poor with a congruence of only 11%. FET could clearly separate tumor from background more definitively than when using rCBV perfusion maps. The mean distance between the local hot spots also differed considerably by 25.4 ± 16.1 mm. The authors concluded that FET and rCBV yield different biological information. In another hybrid FET PET/MRI study, FET parameters were compared with DSC perfusion in 32 patients with gliomas [139]. Although quantitative tumor volumes were correlated, there was persistent spatial incongruence in the assessed mixed population of glioma patients. However, simultaneous assessment of tumor using both FET PET and DSC perfusion offers complementary information of imaging biomarkers, specifically tumor vascularity and tumor metabolism. In cases where susceptibility abnormality from prior hemorrhage or surgery precludes assessment of certain regions on DSC perfusion, other sequences including FET PET can overcome this limitation.

Gottler et al. also employed hybrid FET PET/MRI in the clinical work-up of glioma patients to study the inter- and intra-lesional variability of static and dynamic FET compared with DSC blood volumetric perfusion parameters [126]. The FET TBR uptake was significantly higher than rCBV and the location of hotspots differed considerably. There was, however, a significant correlation between peak values of rCBV and static FET TBR across all patients. In addition, clear intra-lesional spatial correlation was found between the dynamic FET parameters and rCBV. Additional evidence was presented in favor of multimodal, multiparametric imaging in the management of glioma patients, where both perfusion and FET uptake provide different, yet equally important information regarding tumor biology.

Thirty-two glioma patients underwent hybrid FET PET/MRI with DSC perfusion to address the question of radiation necrosis compared with tumor progression [140]. Multiple parameters were assessed, and again the complimentary nature of FET PET combined with MRI in the same session was reproducible with synergetic effects. When assessed individually, the accuracy of TBR max (94.1%), TBR mean (88.2%), ADC mean (80.4%), Cho:Cr ratio (96.4%), normalized rCBV (89.9%) was less than when all parameters were combined (96.87%). These studies corroborate the superiority of amino acid PET and point the benefit of hybrid advanced MRI for evaluation of malignant brain tumors. In assessment of glioma recurrence, multiparametric FET PET/MRI with dynamic FET parameters again yielded the highest diagnostic accuracy of any individual modality [141].

Hybrid FET PET/MRI was utilized to compare regional tumor FET uptake versus Cho/NAA on MRS in 41 patients [133]. The volume of elevated Cho/NAA ratio was compared with FET volumes, which revealed abnormal FET activity and Cho/NAA characteristics are not always congruent regionally within same tumor tissue and differ in their center of mass by 9 ± 8 mm. None of the parameters used in the study correlated with tumor grade. However, hybrid PET/MRI was employed to compare FET TBR and rCBV DSC perfusion to grade newly diagnosed, untreated glioma in 72 patients [142]. The diagnostic accuracy of FET PET and PWI to discriminate LGG from HGG was similar with highest area under the curve (AUC) values for TBR mean and TBR max. In cases that had increased tumor signal with both methods, local hotspots were incongruent in 78% with a mean distance of 10.6 ± 9.5 mm. Consistent incongruence within the hotspots provides further evidence of complementary tumoral information from each modality. This same group of investigators found that FET PET was superior to DSC rCBV perfusion for detecting progressive or recurrent glioma (76% vs. 52%) [143]. FET TBR max was the only parameter to discriminate treatment-related changes from progressive or recurrent gliomas with an accuracy of 81%. Spatial incongruence between FET and DSC was up to 75% within intra-lesional hotspots, which was confirmed in 80% of the cases with tissue samples.

In a single center study, hybrid FET PET/MRI was used for 26 patients to assess GBM recurrence versus radiation necrosis [46]. Multiparametric, multimodality assessment yielded an accuracy of 93.8% for FET TBR max, 87.5% for FET TBR mean, 90.6% for normalized rCBV mean, 96.9% for MRS Cho/Cr ratio, and 81.3% for ADC mean. Accuracy of both normalized rCBV mean and ADC mean was improved when combined with FET TBR max or MRS Cho/Cr ratio. TBR max or TBR mean with Cho/Cr ratio yielded the greatest accuracy, approaching 97%. The maximum area under the curve was achieved when combining FET TBR mean, DSC rCBV, and MRS Cho/Cr ratio values. Hybrid FET PET/MRI was also utilized to assess for glioma recurrence in 63 lesions suggestive of recurrence in 47 glioma patients in a retrospective analysis [141]. Diagnosis was based on histology in 23 cases and follow-up imaging in 40 cases. Combination of all multiparametric multimodality parameters was superior to any one individual assessment including static PET, dynamic PET, PWI, or DWI. In another study, 32 glioma patients underwent hybrid FET PET/MRI with DSC perfusion to address the question of radiation necrosis compared with tumor progression [140]. Multiple parameters were assessed and again the complimentary nature of FET PET combined with MRI in the same session was reproducible with synergetic effects.

Increasing attention is being given to the assessment of non-enhancing tumor regions [144], highlighting the need for modalities to reliably distinguish between edema, gliosis, and tumor [113]. There is early compelling prognostic data on widening resection margins beyond contrast enhancement to include non-enhancing mass-like signal abnormality in suspected high-grade gliomas [145]. These early findings suggest that advanced imaging modalities which accurately detect non-enhancing tumor will be of growing value. Hybrid FET PET/MRI was utilized to demonstrate that FET metabolic activity significantly exceeds tumor volume compared with MRI contrast enhancing regions with tumor confirmed using stereotactic biopsy [146]. These results were similar to Lohmann et al. who performed FET PET and conventional MRI to show that 43 out of 50 patients (86%) had FET tumor volumes that were significantly larger than contrast-enhanced volumes [147].

A growing body of evidence favoring the use of FET, its extensive safety profile and benefits of PET/MRI in brain tumor imaging leave little reason to exclude this agent as standard of care for brain tumor diagnosis and management.

## Radiogenomics, Radiomics, and Machine Learning

While the previously discussed advanced and complementary imaging modalities provide critical information, this section seeks to address the abundance of data available in imaging beyond what may be visually apparent. Radiomics is an emerging area of translational research associating qualitative and quantitative data from clinical imaging with tissue histopathology, with the goal of affecting clinical management and predicting outcomes. The term radiogenomics applies when these imaging features are associated with genetic changes. Machine learning describes the study and implementation of computer algorithms that automatically improve at a defined task with experience. Deep learning is a subset of machine learning applied through large and multilayered artificial neural networks inspired by the human brain. Given the complexity and large data sets that clinical imaging provides, which may not always be appreciable to the human eye, machine learning is often used as a primary tool in radiomics [148]. These methods provide future pathways and opportunities for highly accurate, non-invasive diagnostic, and prognostic data.

Either IDH1 or IDH2 mutations are a defining classification for adult gliomas, with vast majority of gliomas as IDH1 mutations. IDH1/2 mutant tumors generally having longer progression-free survival and overall survival whether in low-grade glial tumors compared to high-grade GBM. In addition, IDH mutation with chromosome 1p and 19q co-deletion is the molecular signature for oligodendroglioma, all but removing oligoastrocytoma as a histopathologic diagnosis [149]. In addition, epigenetic differences such as O6-methylguanine-DNA methyltransferase (MGMT) promoter methylation in GBM have shown improved response to combination of temozolomide and radiation therapy versus radiation therapy alone [150]. In addition, both MGMT promoter methylation and IDH mutation are independent factors favoring pseudoprogression over tumor progression in high-grade glioma using standard therapies [151].

In both lower grade gliomas and GBM, multiple textural feature analysis shows a range of 72–95% predictive accuracy for IDH mutation with T1 contrast enhancement and FLAIR images providing the most discriminating information. The addition of advanced MR sequences such as diffusion-weighted images including parameters such as fractional anisotropy and diffusion kurtosis as well as DSC perfusion have increased accuracy. Finally, using deep learning methods, IDH mutation accuracy prediction is similar to textural feature analysis at 85–97% with the advantage of bypassing the time-consuming step of post-processing textural radiomic features. 1p/19q co-deletion accuracy was 92% and MGMT yielded 61–83% accuracy [152, 153].

Recent studies have also included FET PET/MRI hybrid in predicting IDH mutation with 93% accuracy when combining standard parametric data from FET PET combined from textural analysis [154]. In another study combining FET PET and MR fingerprinting, a technique quantifying T1- and T2-weighted MR images, Haubold et al. used machine learning to analyze these combined techniques for glioma mutation. They were able to find area under the curve measurements of 75.7% for MGMT promoter methylation, 88.7% IDH mutation, and 97.8% for 1p19q co-deletion [155].

## Conclusion

Developments in advanced neuroimaging are providing new avenues for detection of recurrent disease and delineation of healthy brain tissue from malignant tissue. Advanced MRI techniques are more effective at identifying disease involved tissue in comparison to traditional MRI, but still have limitations. Hybrid PET/MRI, in particular, FET PET, is increasingly being recognized as a highly accurate method that can improve patient outcomes when used for pre-operative planning and post-treatment monitoring. Radiomics and radiogenomics utilize clinicopathologic, genetic, and imaging data to produce accurate non-invasive diagnostic and prognostic information. Utilization of advanced MRI, hybrid PET/MRI, and radiomics present a considerable opportunity to improve patient outcomes in the field of neuro-oncology.
